# Role of tyrosine phosphorylation in sperm capacitation / acrosome reaction

**DOI:** 10.1186/1477-7827-2-75

**Published:** 2004-11-09

**Authors:** Rajesh K Naz, Preeti B Rajesh

**Affiliations:** 1 Division of Research, Department of Obstetrics and Gynecology, Medical College of Ohio, Toledo, Ohio, USA

## Abstract

Capacitation is an important physiological pre-requisite before the sperm cell can acrosome react and fertilize the oocyte. Recent reports from several laboratories have amply documented that the protein phosphorylation especially at tyrosine residues is one of the most important events that occur during capacitation. In this article, we have reviewed the data from our and other laboratories, and have constructed a heuristic model for the mechanisms and molecules involved in capacitation/acrosome reaction.

## Introduction

The process of fertilization is characterized by a series of complex set of events. It involves a species-specific interaction between egg and sperm activating a chain of events that leads to formation of zygote, fetus and finally a baby. However, before a spermatozoon can fertilize an oocyte, it must undergo a cascade of biochemical and physiological changes that facilitates its binding and penetration into the oocyte [1,2]. This time-dependent acquisition of fertilizing competence has been defined as "Capacitation" [3,4].

Capacitation confers upon the spermatozoon an ability to gain hyperactive motility, interact with oocyte zona pellucida (ZP), undergo acrosome reaction and initiate oocyte plasma membrane fusion [1]. Capacitation normally occurs in the female genital tract, however, it can also be achieved *in vitro*. In fact, most of the information about various aspects of sperm capacitation has emanated from *in vitro *studies. Sperm cells can be capacitated *in vitro *by using chemically defined media containing appropriate concentrations of electrolytes, metabolic energy sources, and serum albumin (cholesterol acceptor). Although minor variations exist between these media depending on the mammalian species, most of these media contain bicarbonate, calcium and a macromolecule predominantly serum albumin.

Although capacitation of a sperm cell is required before fertilization virtually in every mammalian species studied, the molecular mechanisms and signal transduction pathways involved in this process are not clearly understood. Capacitation involves an increase in membrane fluidity, cholesterol efflux, ion fluxes resulting in alteration of sperm membrane potential, increased tyrosine phosphorylation of proteins, induction of hyperactivation and the acrosome reaction. Protein phosphorylation represents a very important aspect of capacitation. Recently, many advances have been made in the study of phosphorylation of proteins during capacitation, which we will review in this article.

## Protein phoshphorylation

Phosphorylation of proteins is a post-translational modification event that acts as one of the cell's regulatory mechanisms to control various processes such as cellular growth, cell cycle control, cytoskeleton assembly, ionic current modulation, and receptor regulation [5,6]. In fact in eukaryotic cells, one of the most common mechanisms for regulating protein activity is the addition and/or removal of phosphate groups from serine, threonine, or tyrosine residues of protein moieties. Addition or removal of phosphate groups can induce allosteric modifications resulting in conformational changes in proteins leading either to their activation or inactivation.

Mature spermatozoa are terminally differentiated and specialized cells. They are highly compartmentalized but are devoid of any major transcriptional and translational activity. Therefore one can justify the importance of post-translational modifications such as protein phosphorylation/dephosphorylation in regulating important phenomena such as sperm capacitation, hyperactive motility and acrosome reaction, which are required for the spermatozoon to reach, bind, penetrate and fuse with the oocyte. The phosphorylation/dephosphorylation state of phosphoproteins is controlled by the activity of protein kinases and phosphatases, and the counteracting activities of these kinases and phosphatases provide cells with a "switch" that can turn on or turn off the function of various proteins.

Earlier studies reported the presence of various phosphoproteins, protein kinases and protein phosphatases in mammalian spermatozoa and implicated their role in sperm motility acquisition, capacitation and acrosome reaction [7,8]. Phosphorylation can occur at serine, threonine, and tyrosine residues in proteins. Although both serine/threonine phosphorylation and tyrosine phosphorylation of proteins have been reported in spermatozoa (discussed below), the tyrosine phosphorylation is very important and may be the primary or even the exclusive indicator of a signal transduction pathway in a cell.

## Protein tyrosine phosphorylation in spermatozoa

### 1.Tyrosine phophorylated proteins

Historically, in 1989, Leyton and Saling provided the first evidence for the presence of tyrosine phosphorylation in mammalian spermatozoa namely the mouse sperm [9]. Using anti-phosphotyrosine antibody they identified three proteins of 52, 75, and 95 kDa respectively, in mouse sperm. The 95 kDa protein showed enhancement in immunoreactivity with the antibody after sperm capacitation and interaction with oocyte ZP proteins [9]. In 1991, the second study by Naz and associates identified tyrosine phosphorylation in sperm of several mammalian species including human, rat, rabbit, and mouse. They reported four sets of tyrosine phosphorylated proteins in the molecular weight range of 95 kDa/94 ± 3 kDa (FA-2 antigen), 46 ± 3 kDa, 25 ± 7 kDa and 12 ± 2 kDa, respectively, in human sperm [10] and also identified a protein of molecular identity of 94 ± 3 kDa in mouse sperm, that was reported earlier by Leyton and Saling [9]. However, this protein of 94 ± 3 kDa was not identified in rat and rabbit sperm. Although it needs to be confirmed using molecular cloning and sequencing studies, it seems that 94 ± 3 kDa is not an evolutionarily conserved protein. Using ^32^P metabolic labeling and *in vitro *kinase assays, human sperm was found to have at least seven proteins (200, 112, 104, 48, 42, 31 and 25 kDa) that are phosphorylated and fourteen proteins (122, 105, 95, 89, 73, 62, 48, 46, 40, 33, 30, 28, 25 and 22 kDa) that are autophosphorylated [11]. Further studies showed that the 94 ± 3 kDa and 46 ± 3 kDa proteins are also phosphorylated at ser/thr residues besides phosphorylation at tyrosine residues [12]. The 46 ± 3 kDa protein was found out to be the FA-1 antigen, which has been known to play an important role in sperm-ZP binding [13]. FA-1 antigen also plays an important role in capacitation [14,15]. Treatment of human spermatozoa with an anti-FA-1 monoclonal antibody (mAb) during capacitation reduces tyrosine phosphorylation of both 94 ± 3 kDa and 46 ± 3 kDa (FA-1 antigen) proteins, which indicates cross-talk between these two proteins [11,15].

It has been observed that different compartments of human spermatozoa undergo a specific sequence of phosphorylation during capacitation and upon binding to zona pellucida [16]. In order to establish the link between the different phosphorylated proteins and a specific sperm function, it is necessary to differentially localize the tyrosine phosphorylated proteins in various regions of spermatozoon. The flagellum seems to be the major component of sperm cell that undergoes tyrosine phosphorylation in most species except boar [17]. Immunocytochemistry has been used to localize tyrosine phosphorylated proteins in flagellum of human [10,18,19], monkey [20], hamster [21], rat [22], and mouse [23] spermatozoa. In a study using immunofluorescence, Urner *et al *[23] localized the phosphotyrosine proteins in the flagellum during capacitation, zona pellucida binding and gamete fusion in mouse sperm. They observed that during capacitation there is an increase in the proportion of spermatozoa with phosphorylated proteins in the whole flagellum [23]. The increase in the phosphorylation in the principal-piece precedes that in the mid-piece, and phosphorylation in the principal- piece is the pre-requisite for phosphorylation in the mid-piece region. Upon binding to the zona pellucida, nearly all mouse sperm became progressively phosphorylated in both the principal-piece and the mid-piece regions [23]. A significant increase in phosphorylation with capacitation has also been observed in human spermatozoa but it is localized mainly in the principal piece [18,19].

A study from our laboratory using human sperm revealed that the capacitating conditions and zona exposure increases the degree of tyrosine phosphorylation per sperm cell as well as the number of sperm cells that were phosphorylated, especially in the acrosomal regions of the sperm head [10]. Interestingly, with these changes, there was also a shift in the site of phosphotyrosine-specific fluorescence from the tail regions of non-capacitated sperm to the acrosomal regions of capacitated/zona-exposed sperm cells. In other cellular systems, there are reports indicating a shift in subcellular localization of various proteins after phosphorylation. In human epidermoid carcinoma A431 cells, it has been shown that the binding of epidermal growth factor (EGF) to its receptor rapidly triggers redistribution of phospholipase C-r_1 _from a predominantly cytosolic localization to the membrane-bound activity, followed by phosphorylation at tyrosine residues. Since the acrosomal region of the sperm cell is involved in sperm-zona interaction, the shift in phosphotyrosine-specific fluorescence seems to have a physiological significance [10].

Tyrosine phosphorylation of the sperm flagellar proteins has shown to be related to the acquisition of the hyperactive motility [20,24], which is required for the spermatozoa to penetrate the cumulus and the zona pellucida of the oocyte. In human spermatozoa, the protein A-kinase anchoring proteins (AKAPs) localized on the fibrous sheath, namely AKAP82, its precursor pro-AKAP82, and FSP95 are the most prominent tyrosine phosphorylated proteins during capacitation [18,25]. In hamster sperm, the homolog of mouse AKAP4 has been identified as the major tyrosine phosphorylated protein in the capacitated spermatozoa [26]. In contrast, in the mouse sperm, AKAP4 is phosphorylated at ser/thr residues not at tyrosine residues. So there are species-specific variations in the pattern of tyrosine phosphorylation even for the same protein. Another protein that gets tyrosine phosphorylated during capacitation is CABYR (calcium-binding and tyrosine phosphorylation-regulated protein). It has been localized on the principal- piece of human spermatozoa [27]. It is speculated that the CABYR is involved in cross-talk between tyrosine phosphorylation and Ca^2+ ^in the signal transduction pathway. A 55 kDa tyrosine phosphorylated protein has been linked to motility in bovine spermatozoa [28]. During capacitation of mouse spermatozoa, heat shock protein (HSP)-90, a highly evolutionary conserved molecular chaperone protein, becomes tyrosine phosphorylated [29]. HSP-90 is also tyrosine phosphorylated in human and rat spermatozoa when incubated under conditions that induce capacitation [29].

In boar spermatozoa, capacitation induces tyrosine phosphorylation of plasma membrane proteins, which are believed to initiate binding to the zona pellucida and induce acrosome reaction [30]. It has been shown that capacitation induces tyrosine phosphorylation of three major (27 kDa, 37 kDa and 40 kDa) and three minor (34 kDa, 47 kDa and 55 kDa) plasma membrane proteins [30]. In a later study, two plasma membrane proteins isolated from capacitated boar sperm cells (35 kDa and 46 kDa) showed high binding affinity with zona pellucida [31]. These two proteins are most likely the 34 and 47 kDa proteins identified earlier. Although, phosphorylation of sperm proteins is a key feature of capacitation, it is not clear how tyrosine phosphorylation of these proteins is involved in sperm-zona recognition or interaction, and acrosomal exocytosis. However in a recent study, Asquith *et al *[32] have examined the relationship between protein phosphorylation and the ability of mouse spermatozoa to interact with zona pellucida. They have identified two chaperone proteins namely endoplasmin (erp99) and heat shock protein 60 (hsp60) expressed on the surface of mouse spermatozoa. Both erp99 and hsp60 proteins are tyrosine phosphorylated and are localized on the plasma membrane of sperm head, the region that participates in zona binding. They proposed that "activation" of erp99 and hsp60 proteins by tyrosine phosphorylation during capacitation may trigger conformational changes facilitating the formation of a functional zona pellucida receptor complex on the surface of spermatozoa [32]. On a similar line, two sperm proteins namely, ERK-1 and ERK-2 (extracellular signal-regulated kinases) have been identified to be tyrosine phosphorylated and "activated" during capacitation in human spermatozoa [33].

It is interesting to know that sperm-oviductal epithelial cell interaction *in vitro *modifies both the sperm tyrosine phosphorylation and capacitation. The selective sperm binding to oviductal epithelial cells [34] suppresses tyrosine phosphorylation of sperm proteins in boar [17] and canine [35] spermatozoa delaying capacitation. This oviductal modulation of tyrosine phosphorulation/capacitation may help to synchronize sperm function with the time of ovulation.

In order to understand the molecular basis underlying capacitation, it is very important to characterize phosphoproteins involved in the signal transduction pathways. Although a large number of proteins have been reported to be tyrosine phosphorylated, very few have been characterized so far. Most of the studies at the beginning used specific inhibitors and/or phospho-specific antibodies to delineate phosphoproteins in sperm cell. Mass-spectrometric analysis provides another powerful approach to identify and characterize these phosphorylated proteins. Ficarro *et al *[36] used this approach to identify proteins phosphorylated during capacitation of human sperm. They also investigated the phosphorylation sites in these proteins. They are able to map more than 60 phosphorylated sequences in sperm cell. They also provided evidence for tyrosine phosphorylation of two proteins namely, valosin-containing protein (VCP) and AKAP3 (sperm tail protein) during capacitation [36]. Also, the gene knockout technology is very helpful in delineating phosphoproteins that have a role in the tyrosine phosphorylation cascade. The studies have shown that the targeted disruption of the *Akap4 *gene causes defects in sperm flagellum and motility. In the mice lacking AKAP4 protein, that undergoes phosphorylation during capacitation, sperm numbers were not reduced but the spermatozoa failed to show progressive motility, rendering the male mice infertile [37]. The mice showed defect in fibrous sheath formation indicating that the AKAP4 is a scaffold protein required for the organization and integrity of the fibrous sheath. The sperm motility is lost in the absence of AKAP4 protein because signal transduction and glycolytic enzymes do not associate with the fibrous sheath [37]. It would be interesting to examine the gene knockouts of other proteins to delineate the missing links in signal transduction pathways involved in capacitation/acrosomal exocytosis.

### 2.Molecular mechanisms of signal transduction in sperm capacitation / acrosome reaction

Several studies have correlated the degree of tyrosine phosphorylation with the capacitative state of spermatozoa. Visconti *et al *[38,39] examined the correlation between the capacitative state and protein tyrosine phosphorylation in mouse spermatozoa. They observed a time-dependent increase in the protein tyrosine phosphorylation of a set of specific proteins in the molecular range of 40–120 kDa, which was correlated with the capacitation state of spermatozoa [38]. Later studies reported that the protein tyrosine phosphorylation increases in spermatozoa during capacitation in various species, including hamsters [40,41], cats [42], pigs [43], boar [44], bovine [45,46], equine [47], cynomolgus monkey [20], tammar wallaby and brushtail possum (marsupial species) [48] and human [49,50]. All these studies provide evidence that the protein tyrosine phosphorylation is an important regulatory pathway in modulating the events associated with capacitation.

The increase in protein tyrosine phosphorylation during capacitation has been shown to be regulated by a cAMP-dependent pathway involving protein kinase A (PKA) in sperm of various species including mouse [39], hamster [40], boar [44], bovine [45,46], equine [47], cynomolgus monkey [20] and human [49,50]. cAMP is a ubiquitous and "central" second messenger in all cell types. It may target cyclic nucleotide gated ion-channels, cAMP-activated guanine nucleotide exchange protein, and PKA in different signaling pathways. In sperm, cAMP has been shown to activate PKA, which regulates protein tyrosine phosphorylation. This signaling pathway is unique to sperm. It has been observed that addition of H89, a protein kinase A inhibitor, during capacitation reduces/blocks and addition of cell permeable analog of cAMP, dibutyryl cAMP, increases tyrosine phosphorylation of sperm proteins [51].

PKA is a tetrameric enzyme composed of two regulatory and two catalytic subunits. The activity of PKA is dependent on the activities of adenylate cyclase and phosphodiesterase. In sperm cell, PKA is compartmentalized thus ensuring specificity of function through binding of its regulatory subunit to the AKAP family of proteins. Different types of AKAPs have been characterized from sperm of different species [18,52,53]. PKA although a serine/threonine kinase, induces and causes an increase in tyrosine phosphorylation of sperm proteins, by indirect activation of tyrosine kinases [49]. Such kinases must be highly specific to spermatozoa, since cAMP-dependent tyrosine phosphorylation has not been reported in any other cell type examined to date. Tyrosine kinases can be divided into two classes namely the receptor tyrosine kinases (RTKs) and non-receptor protein tyrosine kinases (PTKs). RTKs are transmembrane proteins having an extracellular ligand binding domain and an intracellular tyrosine kinase domain. The localization of phospholipase C-γ(PLCγ) on the plasma membrane and its tyrosine phosphorylation-dependent activation in mouse spermatozoa [54] and phosphoinositide 3 kinase (PI-3 kinase) activity operating downstream of tyrosine phosphorylation in human spermatozoa [55], indirectly indicate the presence of RTKs. Upon extracellular ligand binding, a RTK is activated and then phosphorylate itself (autophosphorylation) or other proteins. In contrast, PTKs are located in the cytoplasm, nucleus or anchored to the inner leaflet of the plasma membrane.

Presence of various tyrosine kinases (RTKs and PTKs) has been demonstrated in spermatozoa of several mammalian species. These include c-ras in human sperm cells [56], EGF receptor tyrosine kinase in human, mouse, rabbit and rat sperm cells [57] and bovine sperm [58], c-Abl in human sperm cells [59] and p190 c-met tyrosine kinase in human sperm cells [60]. Recently another tyrosine kinase TK-32 have been identified in pig spermatozoa and it has been demonstrated that activation of TK-32 occur concomitant with capacitation [61]. Insulin-like growth factor I (IGF-I) receptor has been identified in human [62], and bovine [63] sperm. IGF-1 receptor has tyrosine kinase activity and its ligand IGF-1 is present in the seminal plasma. The IGF-1 system (IGF-1/IGF-1R and IGF-1 binding protein) may be involved in the signal transduction pathway leading to sperm capacitation and acrosome exocytosis [62]. The *src*-related protein tyrosine kinase, c-*yes *(cellular-yamaguchi sarcoma viral oncogene) has been detected in the human sperm head [64]. The c-*yes *belongs to PTK family. The activity of the c-*yes *kinase depends on cAMP, indicating again that tyrosine phosphorylation of proteins in sperm cell is a result of the cross-talk between the cAMP pathway and tyrosine kinase(s). The protein kinase C (PKC) is also present in mammalian spermatozoa and its role has been implicated in sperm motility and acrosome reaction [65], but its function in capacitation is poorly understood.

#### a. PKA pathway

The sperm proteins also undergo phosphorylation at ser/thr residues. Studies have shown capacitation-associated increase in phosphorylated ser/thr residues in human [12] and hamster [66] spermatozoa. Besides PKA (a major ser/thr kinase), the extracellular signal-regulated protein kinase (ERK1/2), which is also a ser/thr kinase, is present in sperm cell [67]. Earlier studies using anti-phosphoserine and anti-phosphothreonine antibodies indicated an increase in ser/thr phosphorylation of 18, 35, 43, 55, 94, 110 and 190 kDa proteins during capacitation of human sperm [12]. A recent study used antibody against the Arg-X-X- (Phospho-ser/thr) motif to study the PKA-dependent ser/thr phosphorylation in capacitating human sperm cell in combination with the agents that stimulates (dibutyryl cAMP, dbcAMP and 3-isobutyl-1-methylxanthine, IBMX) or inhibit (H89 and Rp-adenosine-3', 5'-cyclic monophosphorothionate, Rp-cAMPS) PKA [68]. A significant increase in phosphorylation of proteins designated as P80 and P105, respectively, was observed. This study showed for the first time that the phosphorylation of Arg-X-X- (ser/thr) motif that is characteristic of PKA substrates increases during capacitation. These ser/thr-phosphorylated proteins could provide a link between the early increase in PKA activity that is followed by protein tyrosine phosphorylation. In another report, two 36 kDa proteins (36 k-A and 36 k-B) were phosphorylated in a cAMP-dependant manner at serine residues, in hamster sperm flagella during an increase in the hyperactivated motility [69]. A ser/thr protein kinase, ecto-cyclic AMP-independent protein kinase (ecto-CIK) has been localized on the outer surface of mature goat spermatozoa [70]. Its substrate MPS (major physiological substrate) has been characterized and localized in sperm cell and is speculated to play an important role in sperm-egg interaction in this species [70].

#### b. MAP Kinase Pathway

There is evidence that the mitogen-activated protein kinases (MAPKs), also known as extracellular signal-regulated kinases (ERKs), are present in spermatozoa and are involved in sperm motility and capacitation [56,67] and acrosome reaction [71,72]. The MAP kinases are ser/thr kinases that are involved in signal transduction pathways in several cell types. Their activity is regulated by a cascade of events, which starts with the activation of p21 Ras that stimulates a ser/thr kinase Raf (MAPK kinase). Raf phosphorylates and activates MEK. MEK (MAPK kinase), which is a dual specificity kinase, phosphorylates ERK1 and ERK2 (p44/p42 MAPK, respectively). The ERK pathway has been shown to be present in fowl [73] and human spermatozoa [71]. In fowl sperm, it is involved in the phosphorylation of axonemal and/or accessory cytoskeletal proteins and in the regulation of flagellar motility [73]. In human sperm, progesterone stimulates the ERK2 (p42ERK) and thus this kinase may have a role in capacitation and acrosomal exocytosis [71].

The MAPK isoform ERK2 [71], the adaptor protein Shc [74] and Ras [56] have been localized in human sperm head indicating that this pathway may be required for regulating protein phosphorylation in sperm. It has been speculated that MAP kinase may phosphorylate proteins that influence protein tyrosine phosphorylation indirectly. Inhibition of MAPK blocks protein tyrosine phosphorylation associated with capacitation.

#### c. Factors That Affect Tyrosine Phosphorylation during Sperm Capacitation

##### a. Cholesterol

The lipid composition of sperm plasma membrane is different from that of somatic cell plasma membrane. The plasma membrane of sperm head has high amounts of cholesterol, which regulates the membrane fluidity and plays an important role of capacitation [75]. The cholesterol from sperm plasma membrane is transferred to high molecular weight proteins such as albumin and high-density lipoproteins present in the oviductal fluid as the sperm cell traverses through the female genital tract.

Cholesterol efflux from sperm plasma membrane is associated with the activation of membrane signal transduction pathways related to capacitation [76]. β-Cyclodextrins (cyclic heptasaccharides) promote cholesterol efflux from mouse sperm plasma membrane that results in an increase in capacitation and protein tyrosine phosphorylation through the cAMP/PKA pathway [76]. cAMP analogues can induce protein tyrosine phosphorylation in the absence of bovine serum albumin (BSA) and can also overcome the inhibitory effect of cholesterol-3-sulphate in medium containing BSA. Rp-cAMPS, an inhibitor of PKA, decreases the degree of tyrosine phosphorylation induced by either BSA or cyclodextrins [50]. These findings strongly indicate an important role of cholesterol efflux in tyrosine phosphorylation, mediated through the cAMP/PKA pathway. Cholesterol efflux can also have an indirect influence on the signaling pathways. An increase in membrane fluidity after Ca^2+ ^efflux can increase the permeability of sperm cell plasma membrane to various ions such as Ca^2+ ^and HCO_3_^-^, which can then affect the downstream signaling molecules.

##### b. Calcium

Ca^2+ ^influx is one of the crucial biochemical events that occur during capacitation. There is abundant evidence to support requirement of Ca^2+ ^for capacitation [77,78] and induction of acrosome reaction [79]. It has been demonstrated that there is an increase in concentration of Ca^2+ ^in sperm cell during capacitation in several mammalian species [80-85]. It has been observed that different species have different requirements for Ca^2+ ^during capacitation. There is also evidence that different aspects of human sperm function such as capacitation, acrosome reaction and ZP binding *in vitro *has distinctive Ca^2+ ^requirements. Experiments have shown that in humans 0.22 mM of Ca^2+ ^ions are needed for capacitation while ≥ 0.58 mM is required for AR and ZP binding [86]. Although micromolar concentration of extracellular Ca^2+ ^is needed to achieve sperm capacitation in the mouse [77], millimolar concentration is required for capacitation in man [78,87].

Contrasting reports exist on the impact of extracellular Ca^2+ ^on tyrosine phosphorylation in spermatozoa. The reports on mouse [38] and human spermatozoa [88] have documented that increasing amounts of extracellular Ca^2+ ^increase tyrosine phosphorylation. In contrast, other studies have demonstrated the opposite effect [18,89], indicating that Ca^2+ ^negatively regulates tyrosine phosphorylation during sperm capacitation. A recent study was conducted by Baker *et al *[90] to investigate the impact of extracellular Ca^2+ ^on tyrosine phosphorylation of human and mouse spermatozoa and delineate the mechanisms by which this cation exerts its regulatory effect. They reported that Ca^2+ ^suppresses tyrosine phosphorylation by decreasing the availability of intracellular ATP [90]. Since there is an increase in the intracellular concentration of Ca^2+ ^during capacitation of sperm of several mammalian species [33], it could be speculated that such changes modulate protein tyrosine phosphorylation to regulate capacitation and acrosomal exocytosis.

##### c. Bicorbonate (HCO_3_^-^)

The requirement of for HCO_3_^- ^in capacitation is well elucidated [91,92]. The exact mechanism by which HCO_3_^- ^regulates capacitation is not clear. However, the HCO_3_^- ^influx has been associated with an increase in intracellular pH observed during capacitation, regulation of cAMP levels, reversible change in the lipid architecture of plasma membrane, and hyperpolarization of sperm plasma membrane.

The presence of a Na^+^/HCO_3_^- ^cotransporter has been demonstrated in the mouse sperm and it plays a significant role in capacitation [93]. An increase in intracellular pH during capacitation is attributed to HCO_3_^- ^influx [94]. However, the role of pH is not clear since an increase in pH in sperm cell does not induce capacitation [95]. HCO_3_^- ^may be involved in stimulation of adenylate cyclase activity rather than modulation of pH [39,96]. HCO_3_^- ^induces rapid changes in plasma membrane lipid architecture and in motility by a cAMP/PKA-dependent pathway in boar spermatozoa [97]. Harrison [97] and Da Ros *et al *[98] have shown the role of HCO_3_^- ^in protein tyrosine phosphorylation in sperm during capacitation and fertilization. It is interesting that HCO_3_^- ^levels are low in epididymis and high in seminal plasma and oviduct [99]. These changes in the concentration of HCO_3_^- ^in the male and female reproductive tracts could play an important role in the suppression of capacitation in the epididymis and the promotion of this process *in vivo *in the female reproductive tract. It is observed that a protein, designated as CRISP-1, that is added to the sperm surface in epididymis is lost during capacitation [100]. Addition of exogenous CRISP-1 to the incubation medium inhibits tyrosine phosphorylation in a concentration-dependent manner, thus inhibiting capacitation and subsequently the acrosome reaction.

A recent study investigated the effect of *in vitro *addition of HCO_3_^- ^on intracellular cAMP production and protein tyrosine phosphorylation in human spermatozoa [101]. The addition of HCO_3_^- ^in the medium resulted in a significant increase in sperm motility as well as several hyperactivation parameters, mediated by an increase in cAMP production and tyrosine phosphorylation of AKAP3. The effects disappeared with the addition of 4,4'-diisothiocyanostilbene-2, 2'-disulfonic acid, which is a known inhibitor of bicarbonate transport. It was observed that both tyrosine phosphorylation of AKAP3 and sperm motility were blunted by the soluble adenylate cyclase (sAC) inhibitor 2OH-estradiol. These findings indicate that HCO_3_^- ^stimulates human sperm motility and hyperactivation through activation of sAC and tyrosine phosphorylation of AKAP3, causing an increased recruitment of PKA to AKAP3 [101].

##### d. Reactive Oxygen species (ROS)

Studies have suggested that ROS such as H_2_O_2 _and superoxide anion are involved in the regulation of human sperm capacitation and protein tyrosine phosphorylation [19,102]. Some reports suggest that the ROS action lie upstream from cAMP site in the signal transduction cascade [103], while others believe that ROS is localized downstream from cAMP [88]. In a recent study, Rivlin *et al *[104] suggested that H_2_O_2 _activates adenylate cyclase to produce cAMP, leading to PKA-dependent protein tyrosine phosphorylation. Since adenylate cyclase present in sperm is activated by HCO_3_^-^, they proposed that H_2_O_2 _and HCO_3_^- ^can act in concert or partially substitute each other [104]. However, ROS also has detrimental effects on spermatozoa; while low concentrations of H_2_O_2 _are beneficial, high concentrations can lead to sperm immobilization and death. It has been proposed that the accurate balance of amount of ROS produced and scavenged at any moment determines whether a sperm function will be promoted or jeopardized. Nicotinamide adenine dinucleotide (NADH) and NADPH are also known to promote sperm capacitation [104]. It has been found that exogenous NADPH enhances protein tyrosine phosphorylation in bovine sperm [104] and promote capacitation. NADPH acts as a coenzyme for sperm oxidase that generates superoxide anion, which is later dismutated to H_2_O_2 _by superoxide dismutase. The inhibition of lactate dehydrogenase (LDH)-C4 blocks capacitation of mouse spermatozoa *in vitro *[105]. LDH-C4 is a mammalian testis-specific enzyme and is the only lactate dehydrogenase isozyme present in sperm. LDH-C4 seems to play an important metabolic role in sperm capacitation. The oxidation of NADH with the conversion of pyruvate to lactate by LDH provides ATP necessary for PKA activity [105]. NADH-diaphorase (enzyme that transfer electrons from NADH to an electron acceptor such as 2,6-dichlorophenol-indophenol) plays a role in spermatozoal function through ROS generation. Overproduction of ROS due to high diaphorase activity has been observed in some infertile men [106]. The free radical nitric oxide (NO) generated by spermatozoa has been implicated in sperm function [107]. Non-capacitated human spermatozoa produce low levels of NO, whereas under capacitating conditions, a time dependent increase in NO synthesis has been observed [108]. It has been reported that the increased levels of NO during capacitation modulate cAMP pathway that regulates the downstream protein tyrosine phosphorylation [109]. *In vitro *studies have shown that low concentration of NO enhances the acrosome reaction of mouse [110] and bull [111] spermatozoa, as well as the zona pellucida binding ability of human sperm [112].

##### e. Progesterone

Progesterone has been reported to affect several sperm functions especially capacitation, motility and acrosome reaction [113]. The effects of progesterone on spermatozoa are mediated via progesterone binding sites/progesterone receptor (PR) on the acrosomal membrane [114]. Different types of PRs have been shown to be present on the sperm plasma membrane. These are: plasma membrane Ca^2+ ^channel (PR1), a membrane-associated protein tyrosine kinase (PTK; PR2), and a plasma membrane chloride channel (PR3) [115]. The progesterone stimulates Ca^2+ ^influx in the human spermatozoa through PR1 [114] and voltage-dependent calcium channels (VDCC) [116]. In human spermatozoa, progesterone stimulates tyrosine phosphorylation of sperm proteins [117,118] causing hyperactivation [119] with an increase in cAMP levels [117]. The tyrosine kinase-associated PR (PR2) is responsible for the effect of progesterone on the hyperactive motility and acrosome reaction. PR3 mediates the chloride influx, which takes place during the acrosome reaction [115]. Progesterone also increases the membrane fluidity of human sperm plasma membrane, which is an important event in sperm capacitation and tyrosine phosphorylation [115].

##### f. Gamma-Aminobutyric Acid (GABA)

Recently GABA has emerged as a putative modulator of sperm function. GABA is the most widely distributed inhibitory neurotransmitter in vertebrate central nervous system. Three types of membrane receptors (A, B and C) mediate the inhibitory action of GABA. GABA A-receptor has been identified in human spermatozoa. In a recent study, the *in vitro *effect of GABA was studied on bovine spermatozoa capacitation [120]. It was observed that addition of GABA to the incubation medium results in a concentration-dependent increase in the percentage of capacitated spermatozoa. A significant increase in intracellular Ca^2+ ^and cAMP levels was induced by GABA, and the GABA A-R antagonists, bicuculline or picrotoxin abolished the effect [120]. Thus, GABA seems to induce sperm capacitation through a signal transduction pathway involving Ca^2+^, cAMP and tyrosine phosphorylation.

##### g. Angiotensin II (AII)

AII is known to be present in seminal plasma and it modulates the adenylate cyclase (AC)/cAMP signal transduction pathway [121]. The existence of a class of angiotensin receptors (AT1) has been shown in bovine spermatozoa. In the capacitated sperm, AII AT1 receptors are localized in the head and tail, whereas in non-capacitated cells the receptors are localized only in the tail region [122]. AII significantly stimulates cAMP production in capacitated mouse spermatozoa with an associated increase in protein tyrosine phosphorylation [123].

##### h. Cytokines

Cytokines are a family of polypeptide hormones produced primarily by the cells of the immune system in response to various stimuli including foreign antigens. Although originally identified from the immune cells, the non-immune cells also secrete them. The cytokines can have both positive and negative effects on a variety of cell types and tissues. Cytokines are present in circulation and local genital tract secretions in both men and women [124,125]. The effects of various cytokines on sperm cell have also been studied. It has been observed that cytokines can affect sperm cell motility, capacitation, acrosome reaction, zona-pellucida binding and penetration and embryonic development in positive or negative manner [126]. It has been observed that cytokines namely, interferon-α (IFN-α), interferon-γ (IFN-γ) and tumor necrosis factor-α (TNF-α) have negative effect on sperm motility [126], while interleukin-6 (IL-6) enhances sperm cell capacitation and/or acrosome reaction [127]. However, the mechanisms by which cytokines affect sperm functions are not clear. Identification of cytokine receptors in the sperm cell is an important step in elucidating the mechanism of cytokine action. Very few cytokine receptors have been identified on sperm cells till date. A study in our laboratory, demonstrated the presence of IFN-α and IFN-γ receptors in mouse, rabbit, pig and human sperm [128]. The presence of interleukin-2α (IL-2α) [129], interleukin-2β (IL-2β) [129], insulin like growth factor-1 (IGF-1) [62,63] and c-met (hepatocyte growth factor receptor) [60] receptors on human sperm surface has also been documented. A recent study identified the presence of CX(3)CR1 receptors on human sperm [130]. Fractalkine, a CX(3)C chemokine, is present in fallopian tubes. It might play an important role in maintaining the motility of spermatozoa and their ability to undergo the acrosome reaction when sperm transit through fallopian tubes [130]. However, the effect of various cytokines on tyrosine phosphorylation during capacitation has not been extensively studied. Since many of the cytokines affect capacitation/acrosome reaction and tyrosine phosphorylation is required for these processes, it can be envisaged that these cytokines modulate tyrosine phosphorylation in sperm cell.

## Conclusions

**Figure 1:**
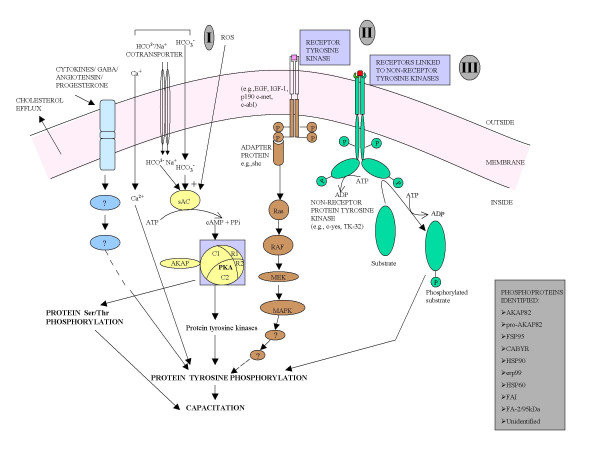
**Heuristic model showing the tyrosine phosphorylation signaling pathways in sperm cell involved in capacitation**. There seems to be three major signaling pathways operating in sperm cell, namely cAMP/PKA-dependent pathway (pathway I), receptor tyrosine kinase pathway (pathway II), and non-receptor protein tyrosine kinase pathway (pathway III). The pathway I, that is exclusive only to sperm cells, seems to be the major pathway among the three pathways. These cascades may not be mutually exclusive and include cross-talk among several molecules. Many key molecules and receptors are still to be identified to completely elucidate the molecular mechanism and signal transduction cascade involved in capacitation. G-protein coupled receptor pathway has not been included in this model.

Before fertilization can occur, spermatozoon undergoes a series of changes to acquire the ability to bind and penetrate the oocyte. These events are regulated by the activation of intracellular signaling pathways involving various molecules such as cAMP, protein kinase A, receptor tyrosine kinases, and non-receptor tyrosine kinases. A number of molecules have been identified, that regulate these pathways such as calcium, bicarbonate, ROS, GABA, progesterone, angiotensin, and cytokines. Studies have shown that phosphorylation of sperm proteins is an important aspect of capacitation and has been shown to be associated with hyperactivated motility, zona pellucida binding and acrosome reaction. Extensive research has started to elucidate various pathways involved in protein phosphorylation during sperm capacitation. Presence of three major pathways involving cAMP/PKA, receptor tyrosine kinases, and non-receptor protein tyrosine kinases have been shown. These pathways are not mutually exclusive and may involve cross-talk among several molecules. The exact pathways have not been clearly delineated. Several questions such as how does the stimulation of cAMP/PKA pathway upregulate tyrosine phosphorylation are still to be answered. Many components of these pathways and several phosphoproteins remain to be identified. The novel technologies such as gene knockout technique will help us to elucidate the key molecules in these pathways. Based upon the present data, we have constructed a heuristic model that is shown in figure 1.
